# Editorial: 46,XX differences of sex development (DSD) outside congenital adrenal hyperplasia (CAH)

**DOI:** 10.3389/fendo.2024.1523964

**Published:** 2024-11-28

**Authors:** Antonio Balsamo, Anu Bashamboo, Silvano Bertelloni, Anna Lauber-Biason

**Affiliations:** ^1^ Alma Mater Studiorum, University Hospital S.Orsola Malpighi, Bologna, Italy; ^2^ Human Developmental Genetics Unit, Centre national de la recherche scientifique (CNRS) UMR 3738, Institut Pasteur, Paris, France; ^3^ Department of Pediatrics, University Hospital Pisa, Pisa, Italy; ^4^ Section of Medicine, Faculty of Science and Medicine, University of Fribourg, Fribourg, Switzerland

**Keywords:** non CAH, 46,XX DSD, testicular/ovotesticular 46,XX, DSD, POI, MRMKS, glucocorticoid resistance, CYP19A1 deficiency

“Differences of sex development” (DSD) are a continuum of congenital conditions with discordance between chromosomal, gonadal, and/or anatomical sex. Based on the chromosomal complement DSDs could be as 46,XY, 46,XX or abnormal chromosomal asset, as 45,X/46,XY (1, 2). There is a large body of literature reviewing 46,XY DSD (3) which is not the case for 46,XX DSD. This, in part, is due to relative lack of information on genes and regulatory networks associated with human ovary development and function.

The most common cause of DSD in 46,XX newborns is congenital adrenal hyperplasia (CAH) due to 21-hydroxylase deficiency, resulting in primary adrenal insufficiency and androgen excess. This disease and other CAHs with androgen excess have been extensively revised in recent literature (4). Thus this Research Topic features clinical aspects, genetics, puberty and fertility outcomes associated with rare forms of 46,XX DSD, outside of CAH.

The review by Stancampiano et al. summarizes sex determination and development. The authors highlight specification of the bipotential anlage as well as the organ specific, mutually antagonistic regulatory networks involved in differentiation of the primordium towards male or female gonad. This is followed by a summary of the development of external genitalia and secondary sexual characteristics. An update on the clinical presentation, genetics, puberty and fertility outcomes in rare causes of 46,XX DSD, outside CAH, is discussed.

This is further explored in the reviews by Abalı and Guran and Ferrari et al. Abalı and Guran focused on differential diagnosis and management of disorders with 1) excessive amounts of endogenous androgens, such as primary glucocorticoid resistance (PGCR) and aromatase deficiency; 2) increased exogenous androgen exposure, such as gestational hyperandrogenism and 3) errors of gonad differentiation [testicular (T)/ovotesticular (OT)-DSD, ovarian dysgenesis (OD)]. Ferrari et al. specifically address molecular mechanisms of anomalies associated with the development of testicular tissue in the 46,XX gonads. The authors provide a comprehensive update on genetic factors contributing to the etiopathogenesis of SRY-negative 46,XX DSDs that include insufficient expression of pro-ovarian genes (*WNT4*, *RSPO1*, *NR2F2*) or overexpression of pro-testicular genes (*SOX* family, *DMRT1*, *FGF9*) as wells as the genetic causes where the molecular mechanism is not completely understood so far (*WT1*, *NR5A1*, *NR0B1*). The authors also briefly discuss a potential role of epigenetic mechanisms and mRNAs in embryonic gonad development, indicating their potential involvement in the etiology of 46,XX DSD (5).

The review by Grouthier and Bachelot address the knowledge gap linking the medical data from non-CAH 46,XX DSD to long-term effects of these pathologies. The reasons for this may include the likely loss of the patient to follow-up in adulthood or the absence of precise data on the accurate prevalence of gender dysphoria and gender reassignment. The authors provide an overview of current data on the long-term follow-up of patients with non-CAH 46,XX DSD, by assessing: quality of life, gender identity, fertility and sexuality, global health, bone and cardiometabolic effects, cancer risk, and mortality.

The review by Federici et al. presents an update on clinical findings and on the principal X-linked and autosomal genes involved in syndromic and non-syndromic forms of Primary ovarian insufficiency (POI). The authors address the heterogeneity of clinical presentation and the genetic etiology of POI. The state of art on the management of the premature hypoestrogenic state as well as on fertility preservation in subjects at risk of POI is discussed.

The review by Herlin presents the advances in our understanding and management of Mayer-Rokitansky-Kuster-Hauser Syndrome (MRKHS), which is characterized by agenesis or aplasia of Mullerian derivatives. The author explores early familial occurrences and the history and state of art for genomic analysis. The review also highlights the psychological impact of MRKH diagnosis and its effect. Finally, the author provides suggestions for future genetic investigations and discusses potential implications for clinical practice.

In this Research Topic, the contributors have discussed not only the clinical and genetic presentations but also the management and therapeutic interventions, including hormone replacement therapy (HRT) in individuals with 46, XX T/OTDSD, 46,XX ovarian dysgenesis that may present with primary amenorrhea and infertility, POI characterized by loss of ovarian function before the age of 40 years, and malformations of internal genitalia such as MRKHS. Additionally, both non-surgical and surgical interventions for vaginal hypoplasia are reviewed. The contributors also assess the possibility of overcoming absolute uterine factor infertility (AUFI) by acquiring genetic motherhood through gestational surrogacy or achieving both gestational and genetic motherhood after uterus transplantation (UTx) (6).

The non-CAH 46,XX DSD group includes a wide spectrum of conditions, with different etiopathogenesis. In the recent years, technological advances, facilitating genomic analysis, has significantly increased our understanding of the genetic etiology of these conditions. However, there still remains a wide knowledge gap in our understanding of genetics and mechanism(s) of human embryonic gonad specification and/or development, and by consequence the diagnostic yield of 46,XX DSDs. This suggests that a proportion of the unexplained cases could be due to disruption of sex-determining gene regulatory elements and opens up avenues for future research. The papers in this Research Topic provide us the current state of art on disease etiology, management and treatment of these conditions. Uncertainties about the gonadal function and gender outcomes may still make the clinical management of these conditions complicated. Patients should be referred to a specialized Centre, where a trained multidisciplinary team is involved in the management and care of these children/adolescents (and their families), from diagnosis to adulthood. At this regard, the transition from pediatric care to adult medicine remains a hot and unsolved topic, at least in practice and in some Countries. Support groups can play a key role in helping patients and their families by sharing their personal experiences. While we know that in general many individuals with DSDs are well adjusted and have a good quality of life, the others may express anxiety and distress related to their condition or report a poor quality of life. We do not have sufficient data to explain the magnitude to which these considerations apply specifically to individuals with non-CAH 46,XX DSDs. To achieve the holistic care and management of individuals presenting with these conditions, it is imperative to improve our knowledge of diagnosis, management and long-term prognosis where clinical care is integrated with psychosocial development and adaptation.
